# Induction of Apoptosis by Metabolites of Rhei Radix et Rhizoma (Da Huang): A Review of the Potential Mechanism in Hepatocellular Carcinoma

**DOI:** 10.3389/fphar.2022.806175

**Published:** 2022-03-02

**Authors:** Huanyu Jiang, Wuyinuo Tang, Yang Song, Wei Jin, Quanyu Du

**Affiliations:** ^1^ Department of Geriatrics, Hospital of Chengdu University of Traditional Chinese Medicine, Chengdu, China; ^2^ Emergency Department, Hospital of Chengdu University of Traditional Chinese Medicine, Chengdu, China; ^3^ Department of Endocrinology, Hospital of Chengdu University of Traditional Chinese Medicine, Chengdu, China

**Keywords:** hepatocellular harcinoma, apoptosis, emodin, rhein, physcion, aloe-emodin, gallic acid, resveratrol

## Abstract

Liver cancer is a global disease with a high mortality rate and limited treatment options. Alternations in apoptosis of tumor cells and immune cells have become an important method for detailing the underlying mechanisms of hepatocellular carcinoma (HCC). Bcl-2 family, Caspase family, Fas and other apoptosis-related proteins have also become antagonistic targets of HCC. Da Huang (Rhei Radix et Rhizoma, RR), a traditional Chinese herb, has recently demonstrated antitumor behaviors. Multiple active metabolites of RR, including emodin, rhein, physcion, aloe-emodin, gallic acid, and resveratrol, can successfully induce apoptosis and inhibit HCC. However, the underlying mechanisms of these metabolites inhibiting the occurrence and development of HCC by inducing apoptosis is complicated owing to the multi-target and multi-pathway characteristics of traditional Chinese herbs. Accordingly, this article reviews the pathways of apoptosis, the relationship between HCC and apoptosis, the role and mechanism of apoptosis induced by mitochondrial endoplasmic reticulum pathway and death receptor pathway in HCC and the mechanism of six RR metabolites inhibiting HCC by inducing apoptosis.

## Introduction

Liver cancer is one of the five most common malignancies worldwide and comprises the second leading cause of cancer-related deaths, with an increasing incidence rate (Marengo et al., 2016). Hepatocellular carcinoma (HCC) accounts for 80–85% of liver cancer cases, and its frequency of occurrence varies greatly from region to region. Most new cases occur in East Asia and southern Africa (Lafaro et al., 2015). Without early detection, HCC patients face extremely low survival rates and limited treatment options with high costs (Gravitz 2014; Orcutt and Anaya 2018). During the past decade, both pharmacologic and nonpharmacologic treatments for HCC, such as resection, suppression, transarterial chemoembolization, and ablation, have improved and become widely used. Small-molecule targeted agents, monoclonal antibodies, and other medicines have gradually been refined (Jindal et al., 2019; Chen et al., 2020). Recently, the role of insufficient apoptosis in the development and progression of some cells, including hepatocytes, has become the primary focus in detailing the underlying mechanism and potential treatment targets of HCC.

As a natural barrier inhibiting the development of cancer, apoptosis is a programmed cell death mechanism that is finely regulated at the genetic level, leading to the effective elimination of damaged cells (such as DNA-damaged or infected cells) (Fuchs and Steller 2011). However, the evasion of and resistance to apoptosis are hallmarks of cancer cells, often leading to chemotherapy failure (Hanahan and Weinberg 2011; Cai et al., 2004). Therefore, a therapeutic strategy for apoptosis-resistant molecules may be effective.

RR comprises the dried roots and rhizomes of *Rheum palmatum* L., *Rheum tanguticum* Maxim. ex Balf., or *Rheum officinale* Baill. According to traditional Chinese medicine theory, RR eliminates heat toxicity, thereby eliminating stagnation and stasis. The most commonly used variety is *R. officinale* (Q. Huang et al., 2007). The main active metabolites of RR are anthraquinone derivatives. The 2020 edition of the Chinese Pharmacopoeia considers the contents of emodin, rhein, emodin, aloe-emodin, and chrysophanol in dried RR as quality control standards. Total anthraquinone content was calculated using these five metabolites. Some of these anthraquinone derivatives have attenuated HCC through apoptosis.

## Search Strategies

**FIGURE 1:**
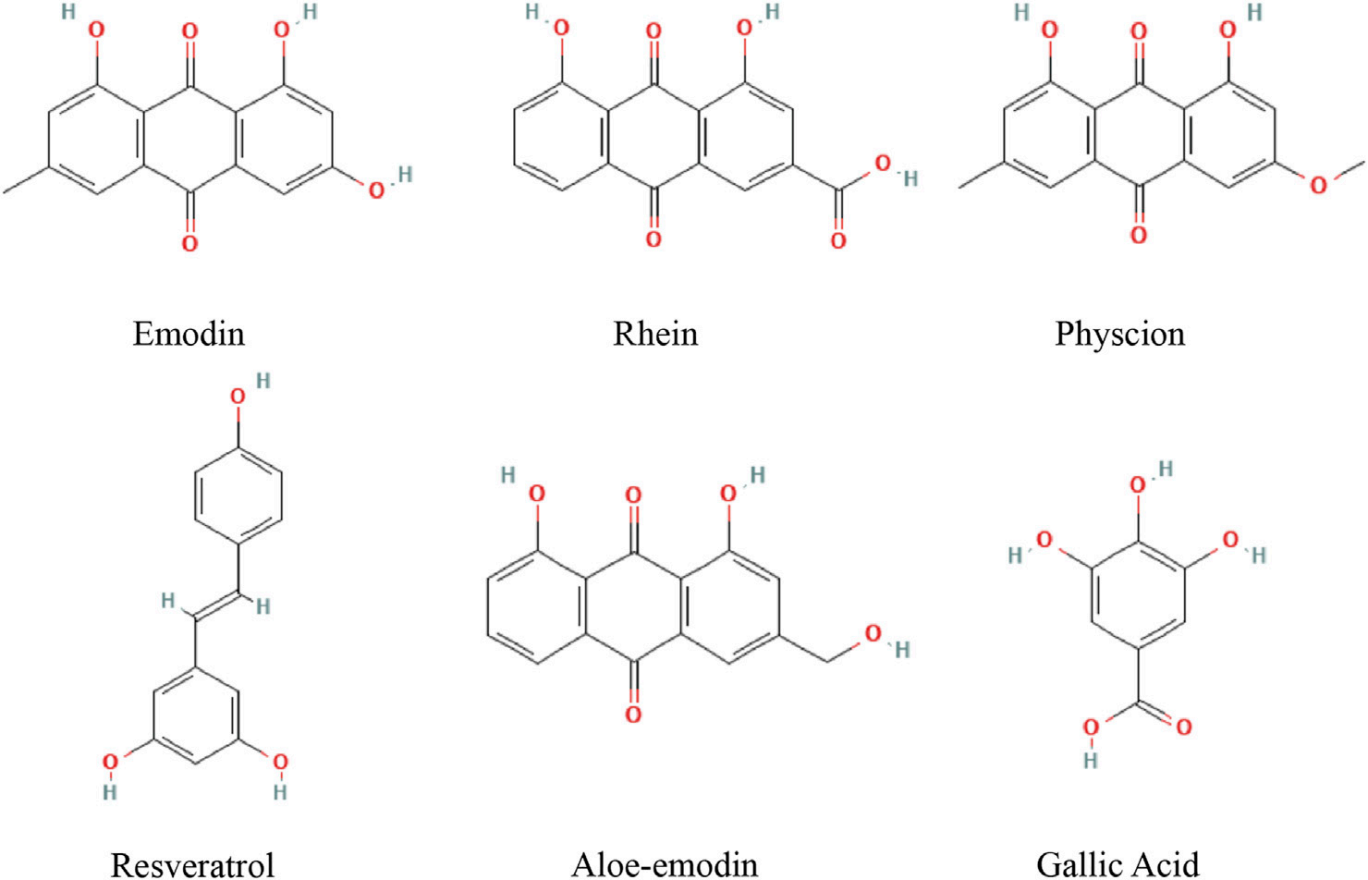
Chemical structures of active metabolites of RR.

For this review, research articles on the treatment of HCC with active ingredients of RR were collected from PubMed, the Cochrane Library Web of Science, and the EMBASE database. According to the quality control standard of RR in Chinese Pharmacopoeia and the quality evaluation index of RR in the articles, the relationship between 13 active ingredients and apoptosis in HCC was searched in the databases. Such as emodin, rhein, aloe-emodin, physcion, chrysophanol, sennoside B, sennoside A, gallic acid, catechins, resveratrol, epigallocatechin gallate, epicatechin and epicatechin gallate. Active metabolites that is not related to apoptosis were excluded. And the following types of article were excluded from our review: studies not lacking scientific value and those with obvious methodological errors. Overall, we have systematically explained the pharmacological mechanism of emodin, rhein, physcion, aloe-emodin, gallic acid, resveratrol regulating apoptosis in HCC cells, providing a reference for future research (Figure1).

## Apoptotic Pathway

Apoptosis signals are mainly conducted through extrinsic and intrinsic apoptosis pathways (Pistritto et al., 2016). Intrinsic apoptosis mainly includes mitochondrial-dependent pathway and endoplasmic reticulum-dependent pathway. Intrinsic apoptosis is comprised of caspase-dependent apoptosis and caspase-independent apoptosis. Extrinsic apoptosis is mostly initiated by the binding of death receptors and ligands (Susin et al., 2000).

Intrinsic apoptosis pathways, which depend on mitochondria, are induced by a variety of highly heterogeneous intracellular stresses. These pathways are regulated by mitochondrial outer membrane permeabilization (MOMP), which can be changed by permeability transition pore complexes (PTPC) and the B-cell lymphoma-2 (Bcl-2) protein family members with pore-forming activity (Green and Kroemer 2004; Vande Walle et al., 2007). Intracellular stress signals, such as oxidative stress (OS), DNA damage, cytosolic Ca2+ overloads, and an accumulation of unfolded proteins in the endoplasmic reticulum, can activate the Bcl-2 protein family members of Bax and Bak, thereby damaging MOMP and releasing toxic proteins. These include cytochrome C (CYTC), second mitochondria-derived activator of caspases (SMAC), high temperature requirement protein A2 (HTRA2), apoptosis-inducing factor (AIF), and endonuclease G (ENDOG), and are usually retained in the mitochondrial intermembrane space (IMS). These proteins are released to the cytosol, causing caspase-dependent and caspase-independent apoptosis (Danial and Korsmeyer 2004). Caspase is a cysteine protease family that contains 14 identified members, which are divided into two subgroups: interleukin 1*β* invertase (ICE) and abnormal cell death gene product 3 (CED-3). CED-3 subfamily, which is divided into initiator and executioner, is a major participant in the process of apoptosis. Caspase−2, −8, −9 and −10, as promoters of apoptosis, activate the executor of apoptosis through self-activation. Caspase−3, −6 and −7 are the executor of apoptosis and can directly degrade intracellular proteins to induce apoptosis after activation by promoter (Yan et al., 2021). Cytosolic CYTC promotes apoptosis by participating with apoptosis protease activating factor-1 (APAF-1) and dATP in forming apoptosomes, which recruit pro-caspase-9 and trigger the cleavage of pro-caspase-3. SMAC and HTRA2 depress caspase inactivation through inhibiting several members of the IAP family, thereby promoting the caspase-9→caspase-3 proteolytic cascade (Slee et al., 1999). AIF and ENDOG induce caspase-independent apoptosis by relocating to the nucleus, where they mediate large-scale DNA fragmentation (Büttner et al., 2007). In addition, HTRA2 can also act on the cytoskeleton because of its serine protease activity (Vande Walle et al., 2007). Endoplasmic reticulum stress (ERS) is the core of endoplasmic reticulum-dependent pathway. ERS refers to the accumulation of misfolded proteins, unfolded proteins, or correctly folded proteins in the endoplasmic reticulum, as well as changes in Ca2+ concentration and cholesterol synthesis, resulting in increased endoplasmic reticulum pressure and disturbance of cell balance (L. Zhang and Wang 2016). The unfolded protein reaction (UPR) triggered by the accumulation of unfolded proteins or misfolded proteins is the primary pathway of ERS. UPR is a self-protective measure for cells to promote endoplasmic reticulum folding ability. It depends on protein kinase R-like endoplasmic reticulum kinase (PERK), inositol requiring enzyme 1 (IRE1), and activating transcription factor-6 (ATF6). UPR can initiate cell apoptosis through signal molecules such as CHOP, caspase-12, JNK, Bax, etc. when cell damage is severe (Metcalf et al., 2020).

The extrinsic apoptosis pathway is a caspase-dependent subroutine of cell death induced by extracellular stress signals that are sensed and propagated by specific transmembrane receptors. When a death receptor is stimulated by its corresponding death ligand, its death domain (DD), a protein–protein interaction domain within the cell, interacts with other DD-containing proteins (Boldin et al., 1995; Schulze-Osthoff et al., 1998; Fulda and Debatin 2003; Guicciardi and Gores 2009). Subsequently, adaptor proteins recruited at the DD of Fas, i.e., a Fas-associated protein with a DD (FADD) or TNFR-associated DD (TRADD), capture other proteins. These may include receptor-interacting protein kinase 1 (RIP1), multiple isoforms of cellular FADD-like interleukin-1 converting enzyme inhibitory proteins (c-FLIP), cellular inhibitors of apoptosis proteins (cIAPs), and pro-caspase-8 and/or -10. These protein combinations form the death-inducing signal complex (DISC), which regulates the activation of caspase-8 (or -10) (Boatright and Salvesen 2003; Chang et al., 2003). Pro-caspase-8 (−10) catalyzes the proteolytic maturation of downstream effectors (e.g., caspase-3, -6, and -7), thereby triggering cell death (Budd et al., 2006; Kuribayashi et al., 2006). Some cells require an expansion step induced by caspase-8. In this case, capase-8 activates the cleavage of the BH3-interacting domain death agonist (BID), thereby generating truncated BID (tBID), which induces MOMP (Plati et al., 2008). Therefore, this works in conjunction with the intrinsic apoptosis pathway.

## Apoptosis in HCC

**FIGURE 2:**
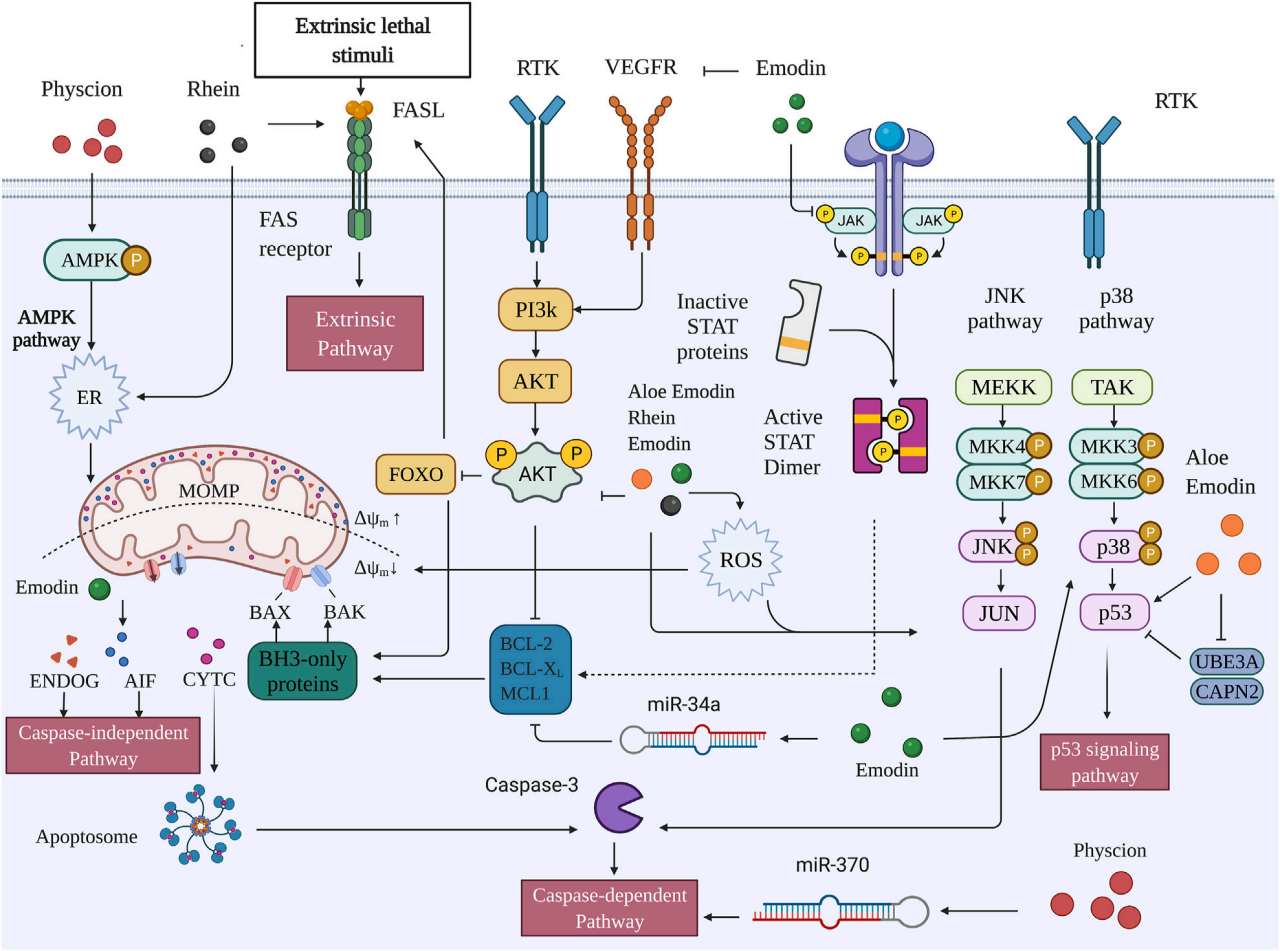
Pro-apoptotic capacities of active metabolites of RR in HCC (Created with BioRender.com).

In normal hepatocytes, apoptosis is finely regulated by a series of genes to maintain homeostasis. Dysregulation of the balance between cell replication, growth, differentiation, and apoptosis is possibly related to the occurrence of preneoplastic lesions and hepatocarcinogenesis Figure 2.

### Mitochondria-Mediated Apoptosis Pathway in HCC

Mitochondria mediated apoptosis is closely related to the Bcl-2 family which Bcl-2 family is divided into three main groups (Youle and Strasser, 2008). The first group is members of the anti-apoptotic family, including Bcl-2, Bcl-XL and Mc1-1, etc. Bcl-2 is usually located in the mitochondrial membrane and endoplasmic reticulum, and inhibits the activity of pro-apoptotic proteins and prevents MOMP by binding to the domain of pro-apoptotic protein BH3. The second group is pro-apoptotic family members, mainly including Bax, Bak and so on. Bax mostly exists in cytoplasm in the form of monomer, and after receiving apoptotic signal stimulation, Bax is transferred to the outer membrane of mitochondria, where Bax and Bak play a pro-apoptotic role together. A decrease in Bcl-2 leads to the fall of Bcl-2/Bax ratio, which is considered as a reliable signal of apoptosis. The third group is BH3-only pro-apoptotic family members, including Bad, Bid and BNIP3, which can induce apoptosis by blocking Bcl-2 or cooperating with Bax (Neophytou et al., 2021).

The expression of anti-apoptotic and pro-apoptotic members of Bcl-2 family is different at different stages of HCC. Most studies have shown that the expression of anti-apoptotic members such as Bcl-2 and Bcl-XL is elevated in tumor tissues of HCC patients (Hosseini-Khah et al., 2021; Y. YangZhu et al., 2011; Watanabe et al., 2004). Also, their elevation showed a significant anti-apoptotic effect on HCC cells such as HCC-9204 and SMMC-7721 *in vitro* (L. Yang et al., 2002; Yao et al., 2012) The decrease of Bax, Bad and other pro-apoptotic members in HCC tissues is associated with adverse clinical features such as vascular invasion, tumor differentiation and AFP (Hu et al., 2015; Garcia et al., 2002). Other studies have shown that Bcl-2 and Bcl-XL are expressed in a lower level in HCC than in normal liver tissues (X.Z. Guo et al., 2002). A research found that Bcl-2 expression was not even observed in HCC tumor tissues (Yoon et al., 1998). This is consistent with more active apoptosis in HCC cells than in normal liver tissues. Lower levels of Bcl-2 may inhibit the growth of tumors, and may also clear senescent tumor cells and promote the development of HCC. In conclusion, the Bcl-2 family tree changes in different tissues and stages of HCC. Interestingly, the expression of Bax decreased in different situations where Bcl-2 was increased or decreased. When Bcl-2 is lowered, Bax dropped more drastically, leading to the change of Bcl-2/Bax (X.Z. Guo et al., 2002). Changes in the ratio of anti-apoptotic members Bcl-2/MCL-1 also have an important impact on the efficacy of anti-tumor drugs in HCC (Tutusaus et al., 2018). Therefore, in mitochondria mediated apoptosis pathway, the balance of anti-apoptotic protein and pro-apoptotic protein is vital in the occurrence and development of HCC.

The anti-apoptotic mechanism of HCC is most closely related to MAPK and PI3K signaling pathways. MAPK pathway is an important signal transduction system of eukaryotic cells, which is comprised of four subfamilies. Extracellular signalregulated protein kinase (ERK), C-Jun N-terminal KINse/stress-activated protein kinase (JNK/SAPK), P38 kinase, and ERK5. Activation of ERK can promote the phosphorylation of Bcl-2, inhibit the apoptosis of HCC cells, and promote tumor development. The anti-apoptotic effect of ABT26-3, Gossypol and other Bcl-2 inhibitors is determined by the activation of ERK pathway (B. Wang et al., 2014; X. Zhao et al., 2011). The therapeutic effects of some natural products and anticancer drugs on HCC also involve the inhibition of ERK pathway, reduction of Bcl-2 (Tsai et al., 2020; W.T. Chen et al., 2019). Activation of JNK pathway promote the evasion from tumor surveillance. On the contrary, blocking JNK expression can inhibit the development of HCC cells, increase caspase recruitment, and induce apoptosis (Mucha et al., 2009). The p53 tumor suppressor gene (TP53) is a common downstream target of JNK and MAPK pathways. P53 can rely on the pro-apoptotic members of the Bcl-2 family such as Bax and Bak to send death stimuli to the mitochondria and induce apoptosis. P53 mutation is a common phenomenon in various cancers, and mutated P53 is also exist in HCC. The expression of Bax and Bak decreased significantly after p53 mutation, and the proliferation and metastasis of tumor cells induced by p53 mutation is an important mechanism of HCC (Meng et al., 2014). Bcl-2 and p53 antagonize each other. Bcl-2 can resist the pro-apoptotic effect of wild-type p53 in tumor cells, and the expression level of bcl-2 is increased in various tissues of p53 knockout mice. The induction of apoptosis through the activation of p53/Bcl-2 pathway in HCC cells is the main mechanism some antitumor drugs (Jiang et al., 2015). Coincidentally, the decreased p53 function is accompanied by overexpression of Bcl-xL and MCL-1, as well as a lack of response to Fas owing to the downregulation of FasL and the reduced expression of Bid in HCC associated with hepatitis B or C infections (May and May, 1999).

Phosphoinositide 3-kinase (PI3K) family is a proto-oncogene that regulates both inositol and phosphatidylinositol (PI). PI3K/Akt is an important pathway for regulating apoptosis in cancer cells. PI3k-activated Akt regulate apoptosis through the phosphorylation of downstream proteins mTOR, NF-ΚB, Bcl-2, Bad and Caspase-9 (G. Song et al., 2005). The activation of the PI3K/Akt/mTOR pathway negatively regulates Bad and positively regulates anti-apoptotic Bcl-2 family members, thereby promoting tumor growth and progression (Pollak, 2012). Additionally, the activation of nuclear factor-ΚB (NF-ΚB) upregulates the caspase-8 inhibitors c-FLIP, 191 c-IAP1, and c-IAP2, as well as the anti-apoptotic proteins Bcl-xL and Mcl-1 (Wajant et al., 2003). Activation of Akt in Hep3B increases cell resistance to antitumor drugs. Activation of Bcl-2 can increase the cancer stem cell-like behavior of Hep3B, promoting cell growth and tumor metastasis (You et al., 2017). CD133+ HCC cells contribute to chemoresistance through preferential activation of Akt/PKB and Bcl-2 cell survival response (S. Ma et al., 2008).

### Endoplasmic Reticulum Mediated Apoptosis Pathway in HCC

When ERS is persistent, UPR is the main cause of apoptosis. UPR can initiate apoptosis through signal molecules such as CHOP, caspase-12, JNK, Bax, etc. CCAAT/enhancer binding protein homologous protein (CHOP) is infrequently expressed in normal cells and mostly activated in ERS state. CHOP triggers apoptosis through Inositol Requiring (IRE) 1-CHOP, PKR-like ER kinase (PERK)-CHOP, and activating factor (ATF) 6-CHOP, respectively. In HCC, CHOP can transcriptively up-regulate the death receptor TNF-related apoptosis ligand receptor 2 (TRAIL receptor 2), and then activate the extrinsic apoptosis pathway. In addition, CHOP induces apoptosis by the transcriptional induction of Bim (Yamaguchi and Wang 2004). ATP Citrate Lyase, a critical enzyme that inhibits cancer metabolic reprogramming, induces ERS in HCC cells and promotes apoptosis of HCC cells by activating the P-EIF2α/ATF4/CHOP axis (Zheng et al., 2021) Both PI3K inhibitors and melatonin can increase the expression of CHOP in HCC cells and inhibit the PI3K/Akt pathway to reverse ERS-induced adriamycin resistance (Fan et al., 2013). Silencing the expression of CHOP in HCC cells can significantly reduce ERS-induced apoptosis (Lei et al., 2017).

Caspase-12-mediated apoptosis is unique to the endoplasmic reticulum pathway. Caspase-12 exists in the outer membrane of the endoplasmic reticulum and is a key protein in the endoplasmic reticulum-mediated apoptosis pathway (Vande Walle et al., 2016). Caspase-12 is activated by tumor necrosis factor receptor associated factor 2 (TRAF2), calproteinase, and caspase-7, and induces apoptosis of HepG2 and BEL-7402 cells (Song et al., 2021).

Persistent ERS can also be activated by phosphorylation of JNK through IRE1-*α*. JNK directly regulates Bcl-2/Bax ratio and induces apoptosis by increasing death receptor level and mitochondrial permeability (Shigemi et al., 2017). *In vivo*, mice deficient in both JNK1 and JNK2 have an increased risk of developing HCC (Das et al., 2011). *In vitro*, activation of JNK promotes apoptosis of Hep3B cells (S.Y. Kim et al., 2020).

### Death Receptors Mediated Apoptosis Pathway in HCC

Death receptors associated with HCC apoptosis include Fas, DR5, DR4, DR3 and TNFR1, which exist on the cell surface in the form of membrane molecules and bind with TNF-related apoptosis-inducing ligand (TRAIL) to induce apoptosis. The expression rate of Fas in serum of HCC patients is 100% while it is reduced in poorly differentiated cancer cells. The level of Fas/FasL in cancer cells can be used as a prognostic indicator for HCC patients and predict the recurrence of HCC (Ito et al., 2000; Sacco et al., 2000). The expression of Fas on immune cells also plays an important role in HCC. The interaction of Fas/FasL can lead to excessive turnover of CD8^+^ T cells in HCC patients (C.L. Guo et al., 2014). Elevated Fas expression is related to increased apoptosis of circulating CD8 (+) T cell in HCC patients. This may be an important mechanism of immune escape of HCC cells. Death receptor 5 (DR5) is rarely expressed in normal liver cells, but highly expressed in HCC cell lines. The specific agonistic antibody against DR5 can selectively induce HCC cell apoptosis *in vitro* and is harmless to normal hepatocytes (Zhu et al., 2006). Therefore, DR5 is also a critical target in HCC cell apoptosis induced by a variety of natural drugs and anti-tumor drugs (Kawahara et al., 2013; J; Yang et al., 2011). Death receptor 4 (DR4) and death receptor 3 (DR3) also induce apoptosis in HCC cells. In Hep3B and other HCC cell lines, DR4 is targeted by Mir-106b, and Mir-106b inhibitors can induce increased DR4 expression and enhance TRAIL-mediated HCC apoptosis (Xu et al., 2017). The expression of DR3 increases in HepG2, Huh7, SMMC7721 and BEL-7402 HCC cells. Silencing DR3 in BEL-7402 inhibited the expression of NF-κB and p53, enhanced the expression of Fas, caspase-3 and caspase-8, and induced the apoptosis of HCC cells (Zhang Y. C. et al., 2015). Tumor necrosis factor receptor 1 (TNFR1) has a dual role in the development of HCC. The formation of TNFR1-complex I supports cell survival while TNFR1-complex II leads to apoptosis (Zou et al., 2020).

## Active Ingredients of Rhei Radix et Rhizoma Used for HCC Treatments

### Emodin

Emodin (1, 3, 8-trihydroxy-6-methylanthraquinone) is a natural product derived from several Chinese herbs, including *R. palmatum*, *Polygonum cuspidatum*, and *Polygonum multiflorum*. It comprises an orange-red powder or crystal that exists in the form of its glycosides (X. Dong et al., 2016). Emodin is an insoluble compound with a melting point of 255°C and is soluble in alcohol and dimethyl sulfoxide (Semwal et al., 2021). Emodin directly regulates both intrinsic and extrinsic apoptosis as well as affects upstream pathways such as the MAPK, PI3K/AKT, VEGFR, and miRNA.

Lin et al. have demonstrated that emodin inhibits the proliferation of SMMC-7721 cells and induced apoptosis in a dose- and time-dependent manner and suppressed tumor growth in BALB/c-nu nude mice inoculated with SMMC-7721. Moreover, emodin may exert these effects by activating p38 and inhibiting p-AKT. In addition, emodin mildly suppresses the activation of c-Jun N-terminal kinase (JNK). Given that JNK is a sub-pathway of the MAPK pathway and plays an important role in apoptosis, the JNK pathway may minimally influence emodin’s proapoptotic effect (Lin et al., 2016). Yang et al. incubated BEL-7402 cells with emodin at 25, 50, 100, 200, 400, and 600 μmol/L for 12, 24, and 48 h, and found the emodin successfully regulated intrinsic apoptosis by upregulating the expressions of BAX, CTYC, actin fiber-associated protein 1 (AFAP1), cleaved-caspase-9, cleaved-caspase-3, and Bcl-2. Moreover, the sterol regulatory element binding protein 1 (SREBP1) was also regulated, and a significant decrease in the expression of mRNA and proteins of SREBP1 was observed. Furthermore, apoptosis and similar changes in protein expressions were found in SREBP1 knockout B cells, but the apoptosis rate was lower than that in emodin-treated cells; this suggests that RR induces apoptosis through both SREBP1-dependent and SREBP1-independent pathways (N. Yang et al., 2019). A recent study by Cui et al. indicated that emodin induced intrinsic apoptosis and extrinsic apoptosis in HepG2 cells, in which treatment with 10–100 μM of emodin attenuated the phosphorylation of AKT and ERK and promoted phosphorylation of p38. Notably, inhibiting PI3K/Akt and ERK and activating p38 can strengthen emodin-induced apoptosis (Cui et al., 2016). Similar results were also observed in another study by Bai et al., who studied the mechanism of emodin from the perspective of the VEGF-AKT-ERK1/2 signaling pathway. In their study, emodin reduced p-VEGFR2, p-ERK, p-ERK1/2, and p-AKT levels in both HepG2 cells and BALB/C nude mice subcutaneously injected with HepG2. Additionally, emodin increased the expression level of miR-34a and reduced the protein levels of SMAD2, SMAD4, and p-SMAD2 (Bai et al., 2020). Subramaniam et al. treated HepG2 cells with 10, 25, and 50 μM of emodin and found that emodin induces apoptosis through inhibiting the STAT3 signaling cascade. Specifically, emodin suppressed STAT3 phosphorylation, its translocation to the nucleus, and its binding capacity to DNA in HepG2, which is mediated by modulating the activation of upstream kinases c-Src, JAK1, and JAK2 (Subramaniam et al., 2013). The Hippo pathway is closely related to OS with Yes-associated protein 1 (YAP1) as one of its key downstream targets and a transcriptional activator that mediates reactive oxygen species (ROS) signals. Large tumor suppressor homolog 1 (LATS1) is a signal protein related to the Hippo pathway. (Morinaka et al., 2011; Wada et al., 2011; Xiao et al., 2011). Lee et al. treated HepG2, SK-Hep-1, Huh-7, and HeLa cells with 3–30 μM of emodin and found that low-dose emodin exerted cytoprotective effects by attenuating arachidonic acid and iron-induced OS. Thus, lactate dehydrogenase (LDH) levels and apoptosis induced by OS decreased. In HepG2 cells, emodin induced the phosphorylation of YAP and LATS1, but HeLa cells did not show these changes. In addition, the AMPK marker p-liver kinase B1 (LKB1), which is the upstream target of acetyl CoA carboxylase and AMPK, was upregulated, whereas emodin demonstrated no cytoprotective effect in LKB1-deficient HeLa cells (Lee et al., 2020).

Emodin contains several free phenolic hydroxyl groups that are easily oxidized in air and have poor solubility, low oral bioavailability, and low stability (Liu et al., 2016a). Therefore, researchers are consistently attempting to improve its formulation, so that this compound can more accurately, efficiently, and stably promote apoptosis in HCC cells. Preparing hydrophobic drugs using nanoparticles as carriers increases the uptake by HCC cells and reduces drug resistance. Synergistic drug use can increase drug concentration in target organs, prolong drug action time, and reduce drug dose and toxic side effects in organs that are not the intended target, so that the drug has stronger anticancer effects. Nanoparticles can be internalized by tumor cells directly without the need for biofilm transport (Mei et al., 2013; Y. Guo et al., 2013). Liu et al., 2016a combined heparin and emodin with a nano-molecular carrier polylactic-co-glycolic acid-D-*α*-tocopheryl polyethylene glycol 1,000 succinate (PLGA-TPGS) to form heparin-loaded PLGA-TPGS nanoparticles and emodin-loaded PLGA-TPGS nanoparticles, respectively. Moreover, the researchers found that the two synergistically promote apoptosis *in vivo* and *in vitro*, and inhibit tumor growth *in vivo* (Liu et al., 2016a; Liu et al., 2016b). Dong et al. distributed emodin in a new type of biomaterial N-acetylaminogalactosyl-poly (lactide-co-glycolide)-succinyl-D-a-tocopherol polyethylene glycol 1,000 succinate (GalNAc-PLGA-sTPGS) to form a new type of nanoparticle, EGTPN, and found that EGTPN *in vitro* induced HepG2 apoptosis more effectively than emodin alone (H. Dong et al., 2018). In addition, combining emodin with other drugs is also a way to enhance its anticancer effects. Kim et al. revealed that 120 μM of emodin can enhance the antitumor effects of sorafenib at a low dose (2 μM) in Hep3B, HepG2, and Huh7 cells. Compared with emodin or sorafenib alone, the combination of the two drugs significantly increased the apoptosis rate of HepG2 cells, inhibited cell proliferation, and induced G1 phase arrest. Furthermore, the combination of the two drugs can regulate lipid metabolism in HepG2 and SK-HEP-1 cells and inhibit STAT3 phosphorylation in HepG2 and PLC/PRF5 cells. This group also performed *in vitro* experiments and found that emodin and sorafenib inhibited tumor growth in xenografted HepG2 and SK-HEP-1 mice (Y.S. Kim et al., 2018).

### Rhein

Rhein (4,5-dihydroxyanthraquinone-2-carboxylic acid) is a lipophilic anthraquinone that exists in *R. palmatum*, *Cassia tora* L., *P. multiflorum*, and *Aloe barbadensis* Miller (Zhou et al., 2015). Induction of ER stress by regulating the mitochondrial membrane potential (MMP) and mitochondrial permeability transition (MPT) are the primary mechanisms by which rhein promotes apoptosis in HCC cells.

In a previous study, swelling and leakage of Ca^2+^ were observed in isolated liver mitochondria when treated with 100 and 200 μM of rhein; however, these changes were inhibited by 1 μM cyclosporin A (CsA), an MPT inhibitor. In addition, rhein may also induce the loss of MMP, activate caspase-3, release CYTC, and reduce the production of ATP in HepG2 cells, whereas CsA may weaken these effects (Du et al., 2013). Moreover, treatment with 5–80 μM rhein may decrease the energy metabolism of SMMC-7221 and SMMC-7221/doxorubicin-resistant cells by inhibiting oxidative phosphorylation. However, CsA cannot reverse the inhibition of energy metabolism via rhein (Wu et al., 2019). Rhein can increase caspase-3 activity when combined with doxorubicin, which may be related to the increase in the concentration of doxorubicin in cells (Wu et al., 2020). Upregulation of the BIM gene (an ER stress-induced gene) is one of the key mechanisms of rhein-induced apoptosis in HCC cells. Wang et al. treated HepG2 cells with 75–150 μM of rhein, and apoptosis was enhanced, which was confirmed by the increase in the expression of the BIM gene, the cleavage of caspase-3,7,8, and the level of t-Bid (J. Wang et al., 2015). ROS can activate the JNK kinase, which subsequently phosphorylates its substrate c-Jun, and phosphorylated c-Jun further induces the activation of caspase-3 (Y. Wang et al., 2018). A recent study found that treatment with 50–200 μM of rhein significantly increased ROS in HepG2 cells and Huh7 cells in a dose-dependent manner. N-acetylcysteine, a ROS scavenger, significantly inhibited the pro-apoptotic effect of rhein. These results demonstrated that rhein-induced ROS activated the JNK/Jun/caspase-3 signaling pathway apoptosis *in vitro* (A. Wang et al., 2020).

Koramagazi et al. demonstrated that rhein can also promote apoptosis in normal hepatocytes. In HL-7702 cells, rhein induced caspase-dependent apoptosis by targeting ER stress-related pathways, including glucose-regulated protein 78 (GRP 78), PKR-like ER kinase (PERK), JNK, and the CCAAT/enhancer-binding protein homologous protein (CHOP) (Koramagazi et al., 2016). Li et al. investigated the activation of the intrinsic and extrinsic apoptosis pathways in L02 cells *via* rhein. Specifically, rhein increased ROS, tumor necrosis factor-α (TNF-α), tumor necrosis factor receptor (TNFR), and TRADD, cleaved caspase-3, and reduced MMP and pro-caspase-9 and -3. Notably, the decrease in the levels of the autophagy-related proteins LC3-II and Beclin-1 and increase in the expression of P62 indicated that rhein promoted apoptosis of L02 cells by inhibiting autophagy and decreasing their self-scavenging ability (Li et al., 2019).

### Physcion

Physcion (1,8-dihydroxy-3-methoxy-6-methyl-anthraquinone), also known as parietin, is a natural anthraquinone derivative that has proven antitumor, antibacterial, anti-inflammatory, antioxidant, and lipid metabolism regulation effects (Xunli and Chen., 2019). Physcion mainly interferes with proteases of the intrinsic apoptosis pathway, ER stress, and miRNA.

Pan et al. revealed that physcion can induce ER stress by activating the AMPK signaling pathway, thus resulting in intrinsic apoptosis. In Huh-7 and Bel-7402 cells, physcion promoted the phosphorylation of AMPK and activated ER stress by increasing caspase-12 activation and protein levels of p-PERK, as well as activating transcription factor 6 (ATF6), heavy-chain binding protein (BIP), GRP78, GRP94, p-eukaryotic translation initiation factor 2A (p-EIF2A), and CHOP. The levels of caspase-12 and CHOP decreased following a treatment with compound C (an AMPK inhibitor) (X.P. Pan et al., 2018). Recombinant DNA methyltransferase 1 (DNMT1) is one of the major enzymes responsible for establishing and maintaining DNA methylation patterns in eukaryotic cells, and is involved in the regulation of miRNAs in tumor cells, including miR-370; by comparison, Sp1 is an upstream transcription factor that regulates the expression of DNMT1 (Datta et al., 2008; Zeng et al., 2012; Yie et al., 2015; Zhao et al., 2015). In another study, physcion increased the level of miR-370, and HCC cell lines transfected with miR-370 mimics showed higher miR-370 levels and apoptosis, whereas an miR-370 inhibitor abolished the physcion-induced apoptosis. Additionally, physcion upregulated pAMPK/tAMPK and downregulated the levels of DNMT1 and Sp1. Accordingly, physcion induced intrinsic apoptosis by upregulating miR-370 *via* the AMPK/Sp1/DNMT1 signaling pathway (X. Pan et al., 2016).

### Aloe-Emodin

Aloe-emodin (1,8-dihydroxy-3-hydroxymethyl-anthraquinone), a common anthraquinone component derived from *Cassia occidentalis*, *R. palmatum*, *Aloe vera*, and *P. multiflorum*, exerts a wide range of pharmacological impacts, including antiviral, anti-inflammatory, antibacterial, anti-parasitic, neuroprotective, and liver-protective effects (X. Dong et al., 2020). The effect of aloe-emodin on apoptosis is closely related to p53 and OS.

In p53 positive HepG2 cells, 1–20 μM of aloe-emodin led to the accumulation of p53 as well as stimulated an increase in the expression of p21 (a cyclin-dependent kinase inhibitor), which was associated with cell cycle arrest in the G1 phase, cell surface molecule lefas/APO1, and Bax. In contrast, aloe emodin did not mediate the expression of Fas/APO1 or inhibited cell cycle progression in P53-deficient Hep3B cells. However, it did promote apoptosis by enhancing the expression of P21 and Bax. (Kuo et al., 2002). Calpain 2 (CAPN2) is a growth promoting protein, and ubiquitin protein ligase E3A (UBE3A) is an oncogenic associated protein, and is a member of the ligase family of E6AP. Both proteins participate in the degradation of p53 (Y. Huang and Wang 2001; L. Liu et al., 2008; Baron et al., 2006; Scheffner et al., 1993; Cooper et al., 2003). A study by Jeon et al. indicated that aloe-emodin can promote intrinsic apoptosis by improving DNA fragmentation and ROS accumulation, and by reducing CAPN2 and UBE3A levels (Jeon et al., 2012). Lu et al. focused on the potential mechanism underlying the pro-OS and pro-apoptotic effects of aloe-emodin. They found that treatment with 10–40 μM of aloe-emodin significantly increased the oxidation of peroxiredoxin (PRDX), a marker of OS and antioxidant in liver tissue. It also increased intracellular ROS levels and decreased glutathione (GSH)/oxidized glutathione (GSSG) in HepG2 cells. Furthermore, a delayed and sustained phosphorylation of JNK and its downstream substrate c-Jun and a decreased phosphorylation of ERK occurred 3 h following aloe-emodin treatment. In addition, HepG2 cells treated with overexpressed antioxidant sod1 (pEGFP-c3/sod1) and aloe-emodin at the same dose decreased OS, JNK activation, and caspase-9. The same treatment was also performed on HCCM cells and Hep3B cells; the results showed that these effects of aloe-emodin were not cell line-specific (Lu et al., 2007).

### Gallic Acid

Gallic acid (3,4,5-trihydroxybenzoic acid, GA) is a naturally occurring phenolic acid. It is widely found in a wide range of plants such as gallnuts, teas, grapes and RR (Ma et al., 2003). GA induce apoptosis in a variety of malignant tumors. GA can selectively induce apoptosis of HCC cell lines without affecting normal hepatocytes. Its mechanism of promoting apoptosis is most closely related to the mitochondrial pathway (Ma et al., 2003).

Sun treated SMMC-7721 cells and hepatocytes with 0, 6.25, 12.5 and 25.0 µg/mL GA and found GA increased the activity of caspase-3, caspase-9 and ROS, decreased MOMP, and induced apoptosis of SMMC-7721 cells. However, GA had no effect on apoptosis of hepatocytes HL-7702. These results indicate that GA has a selective anticancer agent that induces apoptosis in SMMC-7721 cells (Sun et al., 2016). Lima et al. demonstrated a dose-dependent decrease of vitality in GA-treated HepG2 cells and a significant increase of the percentage of cells in early apoptosis. Furthermore, the levels of IL-10 and IL-12 were significantly increased and the levels of IL-8 were decreased after GA treatment. These results suggest that GA has anti-proliferation, pro-apoptotic and anti-inflammatory effects on HepG2 cells (Lima et al., 2016). Shi et al. found GA promoted apoptosis of HepG2 cells and BEL-7402 cells in a dose-dependent manner by up-regulating Bax and down-regulating Bcl-2 and Bcl-XL. Long non-coding RNA (LncRNA) is considered to be a key regulator of tumorigenesis. Overexpression of metastatic lung adenocarcinoma transcript 1 (MALAT1) promotes the proliferation and metastasis of HCC through the MALAT1/Wnt signaling axis. MALAT1 was down-regulated in both GA-treated HCC cells, and overexpression of MALAT1 partially reversed GA-induced inhibitory proliferation and metastasis, and successfully eliminated the inhibition of Wnt/β-catenin signaling. These results suggest that the potential mechanism of GA promoting HCC apoptosis may be related to the inhibition of lncRNA Malat1-Wnt/*β*-catenin signaling axis (Shi et al., 2021). Methyl gallate (MG), a metabolite of GA, can also induce apoptosis of HCC cell lines, which is associated with activation of caspase-3 and regulation of Bcl2, Bax and Bad ligand levels (C.Y. Huang et al., 2021). Like many natural compounds, the clinical application of GA is limited by factors such as low bioavailability, poor oral absorption, and rapid metabolic elimination. Therefore, Ahmed encapsulated GA in PLGA-CS-PEG nanocomposite to form Gallic acid nanocomposite (GANC) and demonstrated that GANC could reduce AFP, ENG, HSP-90, Bcl-2, pro-caspase3 and LCN-2 levels of adult female Wistar rats afflicted with HCC. Pharmacokinetic analysis revealed that GANC displayed a characteristic sustained release profile with 4-fold increase in bioavailability in normal and HCC-induced rats (Ahmed et al., 2018).

### Resveratrol

As a polyhumic compound, resveratrol (3,5,4 ′-trihydroxystilbene) exists naturally in more than 70 plant species (Gecibesler et al., 2021). It can inhibit the proliferation of a variety of human tumor cells, and the potential mechanism of its inhibition on HCC cells is related to the activation of mitochondria-dependent pathway.

Ou et al. found that resveratrol at a concentration of more than 10 μM could significantly inhibit the activity of HepG2 cells and induce apoptosis in a dose-dependent manner via activation of caspase-3 and caspase-9, up-regulation of the ratio of Bax/Bcl-2 and induction of p53 expression. In addition, cell cycle progresssion was arrested in the G1 and S phase. Moreover, the apoptosis-inducing effect of resveratrol can be potentiated by matrine, which is attributed to the cleavage of PARP-1, the activation of caspase-3 and caspase-9, production of ROS and disruption of MOMP (Ou et al., 2014). Karabekir treated HCC male Albino Wistar rats with 50, 75 and 100 mg/kg resveratrol, respectively, and found 50 and 100 mg/kg RSV can significantly decrease enzyme activity (ALT,AST, GGT and ALP). RSV at 75 mg/kg and 100 mg/kg could significantly enhance p53 expression. 100 mg/kg RSV significantly up-regulated Bax, down-regulated Bcl-2, and induced apoptosis of HCC cells (Karabekir and Özgörgülü 2020). Using the same model, Zhang proved that 50 mg/kg RSV can inhibit liver tumorigenesis by inducing cell apoptosis and down-regulating the expression of Myosin Light Chain kinase (MLCK) (X.L. Zhang et al., 2013). Olugbami et al. studied the effects of different doses of resveratrol on HepG2 and their outcomes revealed that resveratrol inhibited the proliferation of HepG2 in a dose-dependent and time-dependent manner. In addition, at lower concentrations (0.39–3.13 μg/ml), resveratrol has higher tendency to activate caspase-3 and caspase-7 (Olugbami et al., 2017).

## Discussion

With advancements of pharmacological technology such as high-throughput screening, an increasing number of natural products with certain chemical structures have exerted a variety of pharmacological effects on patients. As a result, these products have the potential to become new drugs that can be used to treat many diseases. Anthraquinones and their derivatives are widely found in nature and can be divided into three categories. Anthraquinone compounds are the main active metabolites of RR and have a variety of pharmacological activities, including antiviral, anti-inflammatory, antioxidant, and antibacterial effects. Among them, emodin, rhein, physcion, aloe-emodin, gallic acid, resveratrol demonstrate oral bioavailability (OB) values of 24.40, 83.38, 22.29, 47.07, 31.69, and 19.07 as well as drug-likeness (DL) values of 0.24, 0.28, 0.27, 0.24, 0.04 and 0.11 respectively. Moreover, they have superior safety, and other advantages from the perspective of new drug development. The ADME parameters of rhein and aloe-emodin have certain advantages, whereas low bioavailability limits the clinical applications of emodin, resveratrol, and resveratrol; new formulations or structural modifications are needed to improve their pharmacokinetic parameters and promote their clinical applicability. According to current research, emodin, rhein, physcion, aloe-emodin, gallic acid, and resveratrol have considerable therapeutic effects on HCC, with apoptosis playing a critical role in their action mechanisms. Emodin has the most complex action mechanism, as it is related to both intrinsic and extrinsic apoptotic pathways and involves multiple upstream pathways that also interact with each other. Both rhein and physcion are related to the intrinsic apoptotic pathway and interfere with ER stress. The difference is that rhein can also downregulate MMP, whereas physcion can regulate miRNA expression. Aloe-emodin upregulates p53 and promotes OS, indirectly promoting cell apoptosis. Studies on other ways of treating HCC with these constituents, such as anti-angiogenic and anti-metastatic action, cell cycle arrest, and antioxidant and anti-inflammatory activities, are also being performed. These different action mechanisms share some common targets and synergistically provide antitumor benefits (He et al., 2009; Hsu et al., 2010; Zhang K et al., 2015; Xing et al., 2018; Cui et al., 2020).

RR is more commonly used as a laxative in TCM, and anthraquinones also have strong laxative effects (Srinivas et al., 2007). Therefore, it is necessary to watch for gastrointestinal side effects when using anthraquinones. Moreover, problems regarding how to more precisely target the liver, improve efficacy, and reduce side effects remain. There have been attempts to combine with other drugs or nanoparticles, but additional evidence is required (Akkol et al., 2021). Additionally, the current research still focuses on *in vitro* tests, with few animal and clinical tests. The anti-cancer effects of these ingredients *in vivo* still need to be verified. Although many experiments have proven that the active ingredients of RR can regulate various signals in the apoptosis of HCC cells, the specific link between its action mechanism, binding molecules, and binding sites are still unclear. Studies have used rhein and aloe-emodin as ligands and JUN proteins as protein receptors for molecular docking, and found that their affinity values were −6.3 and −6.1 kcal/mol, respectively, indicating strong binding activity between the two and JUN (Jiang L, et al., 2021). A molecular docking of BAX and aloe-emodin showed an affinity value of −15.72, implying a high binding affinity between them (Mulakayala C, et al., 2013). These studies are based on bioinformatic analyses and thus provide predictions for proteins or molecules that may interact with the metabolites of RR, but further experiments are needed to explore specific binding methods and sites.

As the main quality control indicators of RR, emodin, rhein, physcion, aloe-emodin, gallic acid, and resveratrol can inhibit the development of HCC through inducing apoptosis. RR may constitute a potential drug for treating HCC, and its clinical value is worth exploring in future studies.
